# Recovery of erectile function comparing autologous nerve grafts, unseeded conduits, Schwann-cell-seeded guidance tubes and GDNF-overexpressing Schwann cell grafts

**DOI:** 10.1242/dmm.026518

**Published:** 2016-12-01

**Authors:** Florian May, Alexander Buchner, Kaspar Matiasek, Boris Schlenker, Christian Stief, Norbert Weidner

**Affiliations:** 1 Department of Urology, Ludwig Maximilians University, Munich 81377, Germany; 2 Section of Clinical and Comparative Neuropathology, Center for Clinical Veterinary Medicine, Ludwig Maximilians University, Munich 80539, Germany; 3 Spinal Cord Injury Center, Ruprecht Karls University, Heidelberg 69120, Germany

**Keywords:** Erectile dysfunction, Schwann cells, Nerve grafts, GDNF

## Abstract

Dissection of the cavernous nerves during radical prostatectomy for prostate cancer eliminates spontaneous erections. Using the rat as an experimental model, we compared the regenerative capacity of autologous nerve grafts and Schwann-cell-seeded nerve guides. After bilateral excision of cavernous nerve segments, cavernous nerves were reconstructed using unseeded silicon tubes, nerve autografts and silicon tubes seeded with either Glial-cell-line-derived (GDNF)-overexpressing or green fluorescent protein (GFP)-expressing Schwann cells (SCs) (16 study nerves per group). Control groups underwent either a sham operation or bilateral excision of cavernous nerve segments without repair. After 12 weeks erectile function was assessed by neurostimulation and intracavernous pressure (ICP) measurement. The reconstructed nerve segments were excised and histologically analyzed. We demonstrated an intact erectile response upon neurostimulation in 25% (4/16) of autologous nerve grafts, in 50% (8/16) of unseeded tubes, in 75% (12/16) of the Schwann-cell–GFP group and in 93.75% (15/16) of the GDNF group. ICP was significantly increased when comparing the Schwann-cell–GFP group with nerve autografts, unseeded conduits and negative controls (*P*<0.005). In conclusion, Schwann-cell-seeded scaffolds combined with neurotrophic factors are superior to unseeded tubes and autologous nerve grafts. They present a promising therapeutic approach for the repair of erectile nerve gaps.

## Abstract (precis)

**Summary:** Schwann-cell-seeded scaffolds and GDNF-overexpressing Schwann cell grafts are superior to unseeded tubes and autologous nerve grafts for the repair of erectile nerve gaps following nerve excision in rats.

## INTRODUCTION

Neurogenic erectile dysfunction resulting from injured cavernous nerves during surgery still represents a frequent complication after radical prostatectomy for prostate cancer. Whereas current research strategies have focused on pharmacological methods, (e.g. phosphodiesterase type 5 inhibitors) so as to preserve the hemodynamic mechanisms of penile erection, there are no interventions to support cavernous nerve regeneration following radical prostatectomy. Schwann cells (SCs) are the main glia of peripheral nerves and have a key role in nerve regeneration (Gordon and Borschel, 2016; Wang et al., 2015; Jessen and Mirsky, 2016). Adherent molecules on the surface of SCs can secrete extracellular matrix and guide the growth of axons. Neurotrophic factors secreted by SCs might be the most important factors in the microenvironment for regenerating axons (Santos et al., 2016). Growth factors enhance axonal regrowth and promote neuron survival. This regenerative capacity is particularly important in the delayed repair of longer nerve gaps, such as cavernous nerve injury caused by radical prostatectomy. There are various treatment strategies in animal models for the repair of injured cavernous nerves, including mesenchymal stem cells, immunophilins and neurotrophic factors. GDNF has been shown *in vitro* to promote the outgrowth and survival of autonomic nerves including penile erection-inducing autonomic neurons (Palma and Keast, 2006; Laurikainen et al., 2000). Several *in vivo* studies have demonstrated the ability of the GDNF family to enhance functional repair of injured cavernous nerves (Bella et al., 2007; Kato et al., 2007). Therefore, we chose GDNF for this study.

Following nerve injury, SCs might not release enough neurotrophic factors to preserve neuron survival. As neuronal repair mechanisms might take a longer period of several months, previous work has proposed the delivery of growth factors in peripheral nerve repair (Qin et al., 2016). Numerous investigations have demonstrated that cavernous nerves can be successfully repaired using autologous nerve grafts and artificial conduits. The addition of neurotrophic factors and SCs has been shown to further promote nerve regeneration (Xu et al., 2016; Hood et al., 2009).

We previously demonstrated that conduits seeded with syngenic SCs successfully bridge transected cavernous nerves (May et al., 2004). The regenerative capacity can be enhanced by the genetic modification of SCs to overexpress GDNF (May et al., 2008).

The aim of the current study was to investigate and compare different methods of cavernous nerve grafting. Rat cavernous nerve defects were reconstructed by conduits seeded with GDNF-overexpressing SCs. The functional results were compared with those of silicon tubes filled with GFP-expressing SCs, unseeded tubes and nerve autografts.

## RESULTS

**Fig. 1.**
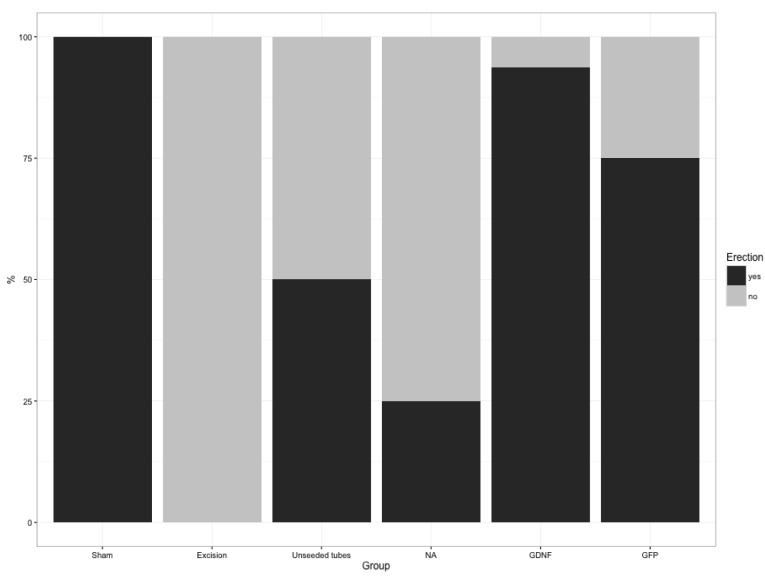
**Recovery of erectile function after bilateral nerve ablation and reconstruction.** At 12 weeks, rats were re-operated and erectile function was evaluated. On direct electrical nerve stimulation, erectile response was analyzed and counted for sham-operated, excision-operated, unseeded tubes, nerve autograph (NA), GDNF-overexpressing-SC-seeded tube (GDNF) and GFP–SC-seeded tube (GFP) treatments.

Achieving a clear, visible erection with a full increase in shaft length on neurostimulation was interpreted as restored erectile function. While all animals of the sham group revealed an intact erectile response, rats after bilateral nerve resection without interposition grafting (control group) showed no inducible erections, confirming that this animal model is reliable (Fig. 1, Table 1).

**Table 1.**
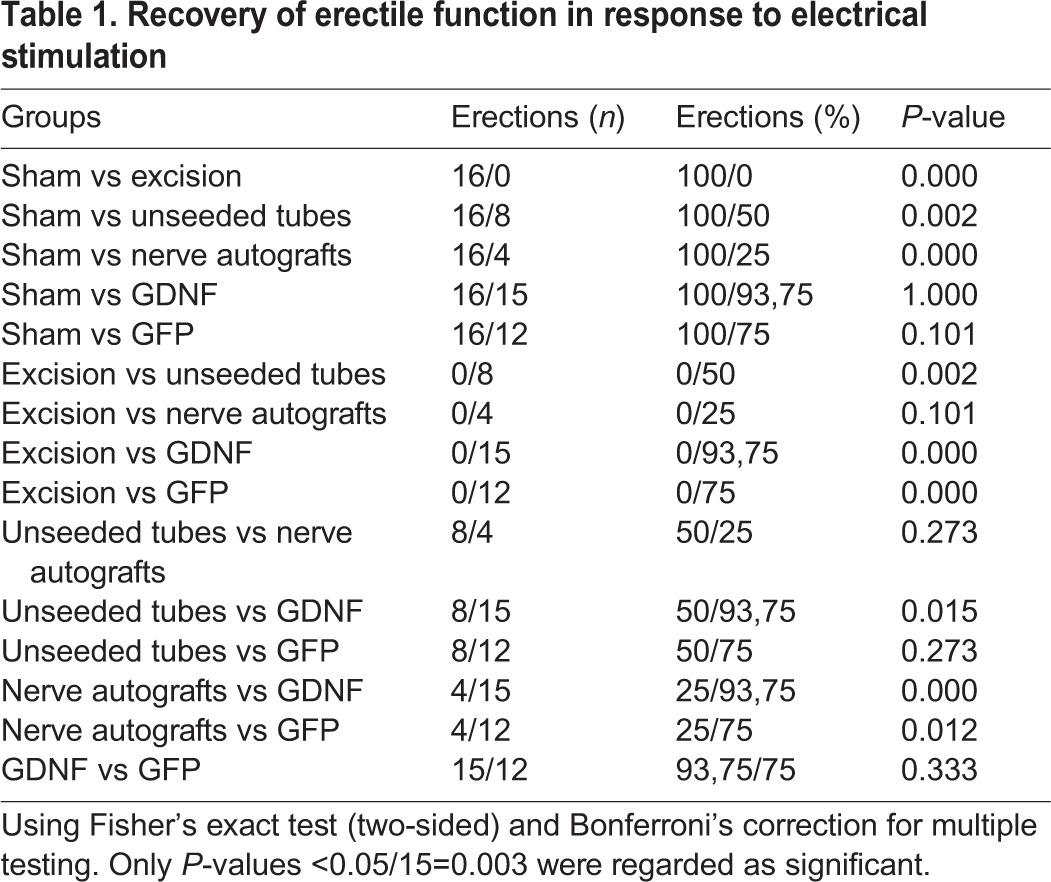
**Recovery of erectile function in response to electrical stimulation**

Neurostimulation led to full erections in 25% (4/16) of rats with autologous nerve grafts, whereas unseeded tubes restored erection in 50% (8/16) of rats with reconstructed nerves (Fig. 1, Table 1). SC-seeded guidance tubes showed the best results, achieving erections in 94% (15/16) of rats in the GDNF and 75% (12/16) in the GFP group. Intact erectile response promoted by GDNF-transduced grafts was significantly superior to nerve autografts (*P*<0.001).

**Fig. 2.**
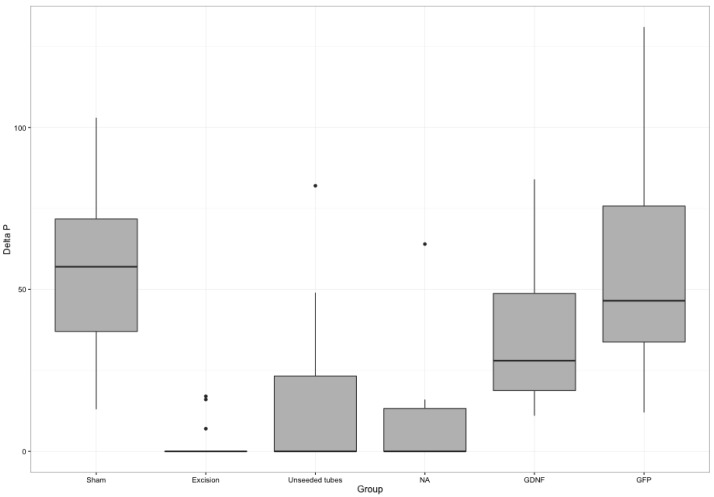
**On direct electrical nerve stimulation, erectile response was quantified by measurement of intracavernous pressure increase.** Values represent mean±standard error of the mean (s.e.m.). The best restoration of this parameter was achieved by GDNF- and GFP-transduced SC grafts (Kruskal–Wallis-ANOVA: all groups *P*<0.001). Both nerve autografts (NA) and unseeded conduits exhibited a significantly lower ICP increase compared with SC-seeded conduits (GFP group).

Neurostimulation with measurement of ICP was used to quantify erectile function. GDNF- and GFP–SC-seeded conduits led to the highest increase of this parameter (Fig. 2, Table 2). ICP was significantly increased comparing the GFP group with unseeded tubes (*P*=0.004), nerve autografts (*P*<0.001) and negative controls (*P*<0.001). Both nerve autografts and unseeded conduit rats exhibited a significantly lower ICP increase compared with the GFP group.

**Table 2.**
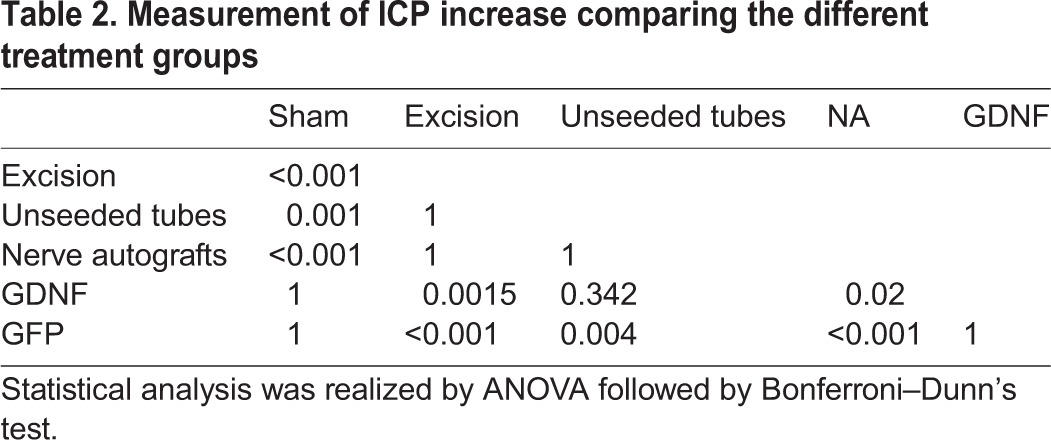
**Measurement of ICP increase comparing the different treatment groups**

**Fig. 3.**
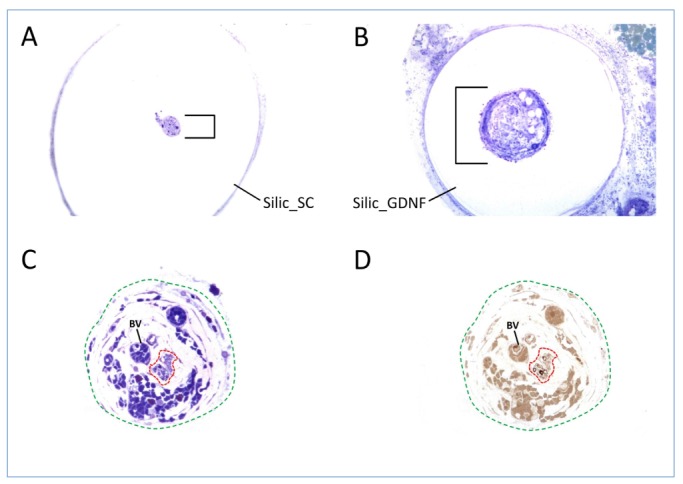
**Microscopic transverse sections of nerve regenerates after 12 weeks.** (A,B) Intratubular regenerates (black brackets) after entubulization with (A) GFP- and (B) GDNF-transduced SCs. (C,D) Detailed histological studies are required to identify regenerating nerve fibers (framed by red dashed line) amongst the entire regenerate (green dashed line) that also is composed of fibrovascular tissue and large blood vessels (BV). Special stains were used to highlight myelinated fibers (D, black rings) within the minifascicles (framed by red dashed line). A,B,C: Azure II Methylene Blue-Safranin; D: p-phenylendiamine.

Histological analysis of the nerve grafts showed that the regenerated nerves were usually localized in the center of the silicon tube encircled by an acellular substance that filled the area between the regenerated nerve and the inner conduit wall (Fig. 3A,B). Special stains show regenerating nerve fibers including myelinated axons within the entire regenerate (Fig. 3C,D).

## DISCUSSION

Recovery of cavernous nerve injury following radical prostatectomy for prostate cancer is often poor despite nerve-protecting techniques. Deliberate excision of the neurovascular bundles for oncological reasons also leads to permanent erectile dysfunction. Autologous sural nerve grafts have been used to repair this injury with insufficient results (White and Kim, 2009). Moreover, they are associated with specific morbidity resulting from a second surgical intervention. Artificial nerve grafts might avoid these deficits.

We therefore looked for alternative nerve-growth-promoting strategies. Using a reliable animal model that leads to complete loss of erectile function unless the nerves are reconstructed, we compared the results achieved by nerve autografts, unseeded silicon guidance channels, SC-seeded nerve guides and conduits seeded with SCs overexpressing GDNF. To our knowledge, this is the first study comparing these modalities for the reconstruction of erectile nerves. This study shows that SC-seeded nerve guides effectively restore cavernous nerve gaps in rodents. We demonstrate that a simple artificial peripheral nerve can be created by placing SCs, and even neurotrophic-factor-overexpressing SCs, within a silicon tube to promote the regeneration of cavernous nerves. We have found that this strategy clearly expands the clinical potential of unseeded tubes, permitting the repair of the majority of injured cavernous nerves. Whereas nerve autografts led to the restoration of erectile function in 25% of grafted nerves, GFP- and GDNF-transduced SC grafts led to success rates of 75% and 94%, respectively. ICP measurement supports these findings showing that the GFP and GDNF group led to the best results, whereas ICP levels were low for the nerve autograft and unseeded tube groups. Histological findings confirm recent data of our group showing that GDNF accelerates cavernous nerve regeneration, enhancing the number and maturation of regenerated axons (May et al., 2008).

Even unseeded conduits led to better results than nerve autografts, in which intraneural scarring might inhibit axonal regrowth. Previous histological findings demonstrated that the architecture of regenerating nerves within silicon tubes often resembles the intact axonal structure in contrast to nerve autografts, which showed only sparse regenerating minifascicles (May et al., 2004). Contrary to artificial delivery systems, SCs are able to react to changes of their environment by secretion of multiple growth factors. A major disadvantage of autologous SCs, however, is the delay caused by culture and purification of SCs before clinical use.

The unique regenerative capacity of SCs declines after longer intervals of denervation. The loss of axonal contact during peripheral nerve damage induces a change from a myelinating to a non-myelinating growth-supportive phenotype with enhanced expression of neurotrophic factors and their receptors (Jessen and Mirsky, 2016; Sulaiman and Gordon, 2013; Wood and Mackinnon, 2015). The upregulation of the so-called regeneration-associated genes (RAGs) is transient and there is a limited time window during which SCs enable axonal regrowth.

Höke et al. (2002) examined the changes in the expression pattern of the GDNF family of growth factors in chronically denervated rat sciatic nerves. Only GDNF mRNA expression was rapidly upregulated in SCs as early as 48 hours after denervation. This upregulation peaked at 1 week and then declined to minimal levels by 6 months of denervation. This study suggests that the limited ability of SCs to support chronically injured neurons with neurotrophic factors might be one of the main reasons for failed regeneration. Therefore, transplantation of gene-modified SCs that produce the needed types of neurotrophic factors represents an effective strategy to overcome this functional deficit.

Several studies provide evidence for the successful use of neurotrophic factor gene therapy in humans. Treatment with adenovirus encoding GDNF, BDNF or transforming growth factor β2 (TGFβ2) significantly prevented the degeneration of facial motor neurons in individuals with facial nerve lesions (Sakamoto et al., 2003). Adenoviral GDNF transfer promoted laryngeal function recovery after recurrent laryngeal nerve injury (Araki et al., 2006) and stereotactic gene delivery for neurotrophic factors was well tolerated in individuals with advanced Alzheimer's (nerve growth factor; Rafii et al., 2014) and Parkinson's disease (neurturin; Marks et al., 2016).

There are major limitations in neurotrophic factor gene therapy for peripheral nerve lesions, as it might provoke uncontrolled and misdirected growth of axons, hypersensitivity and neuropathic pain (Hoyng et al., 2015). Therefore, animal studies must first provide evidence that dose and timing of neurotrophic factor gene delivery is effectively controlled before this strategy can be tested in patients with peripheral nerve injuries.

Adequate axonal guidance for injured peripheral nerves could be accomplished by means of micro- or nanostructured conduits combined with cellular delivery of neurotrophic factors. The supportive effect of these cells might prolong the time window for axonal regeneration and improve the rate of functional restoration even in chronic cases.

## MATERIALS AND METHODS

### *In vitro* experiments

Sciatic nerve fragments from adult male Fischer rats were used for isolation and culture of SCs as previously described (May et al., 2004). Vectors encoding the full sequence of rat GDNF were produced as published by Blesch and Tuszynski (2003). Retroviral vectors expressing GDNF derived from Moloney leukemia virus were used for transduction of SCs *in vitro*. Whereas effective transduction *in vitro* was tested by GDNF-specific ELISA (Promega, Madison, WI, USA), we confirmed *in vivo* GDNF presence by immunhistochemical analysis (May et al., 2008).

We used non-biodegradable silastic nerve guides (length, 5 mm; inner diameter, 0.51 mm; outer diameter 0.94 mm) for interposition grafting. The tubes were filled with the GDNF-SC suspension (cell quantity 25,000 cells/ml) as previously described (May et al., 2008).

### Animal experiments

Forty-eight adult male Fischer 344 rats (250-350 g) were randomized into six groups of eight each (16 study nerves). The bilateral cavernous nerves were transected to create a 5 mm defect, which was immediately reconstructed using unseeded (empty) silicon tubes, nerve autografts, tubes seeded with either GFP- or GDNF-transduced SCs (16 study nerves per group; Table 3). The ipsilateral genitofemoral nerve (7 mm segment) was used for interposition grafting between the transected cavernous nerve ends as previously published (May et al., 2004).

**Table 3.**
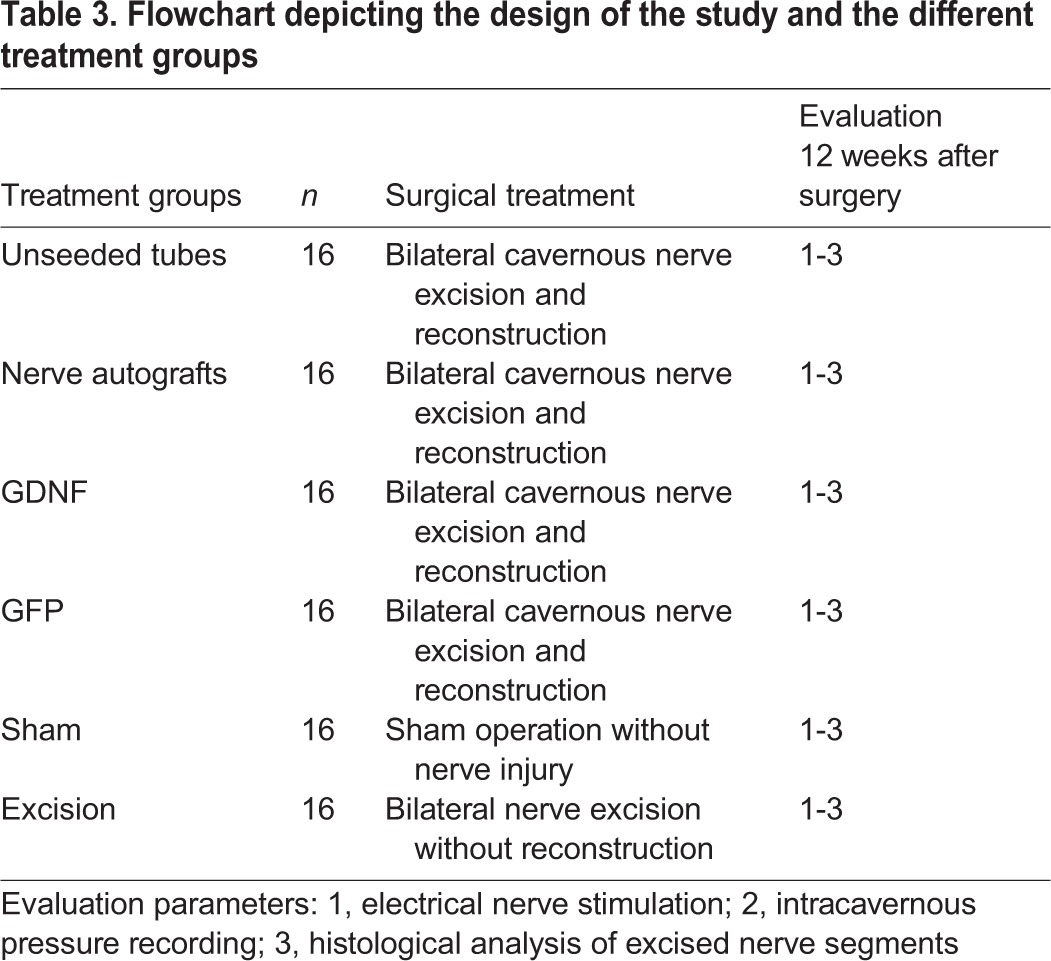
**Flowchart depicting the design of the study and the different treatment groups**

Further animals were either sham-operated or underwent bilateral nerve excision without repair (control groups, 16 study nerves each). The surgical procedures were described previously (May et al., 2004, 2008).

All rats underwent a relaparotomy once after 12 weeks and were euthanized afterwards. Evaluation included neurostimulation of the proximal cavernous nerves over an intact nerve segment and measurement of both intracavernous pressure (ICP) and mean arterial blood pressure (MAP) as described previously (May et al., 2004). All surgical procedures including reexploration and electrostimulation were approved by the local ethics committee and done in full accordance with national and institutional regulations.

#### Histological analysis

The reconstructed nerves were harvested, dissected at mid-regenerate level, fixed and embedded as previously published (May et al., 2008). Semithin sections (0.5 µm) were stained with Azure Blue-Safranin and p-phenylendiamine and then analyzed for regenerating axons and the fascicular formation.

#### Data analysis

Data are presented as mean±s.e.m. Groups are compared by the chi-square and Fisher exact tests. Intracavernous pressure and systemic blood pressure were analyzed by using nonparametric Kruskal–Wallis ANOVA followed by Bonferroni–Dunn's test for individual between-group comparisons at the *P*<0.05 level of significance.
